# Proteomic and Phosphoproteomic Analyses during Plant Regeneration Initiation in Cotton (*Gossypium hirsutum* L.)

**DOI:** 10.3390/genes15081079

**Published:** 2024-08-15

**Authors:** Haixia Guo, Jin Wang, Xuehui Huo, Xiwang Cui, Li Zhang, Xiushan Qi, Xiaoying Wu, Junchen Liu, Aijuan Wang, Jialin Liu, Xiangyu Chen, Fanchang Zeng, Huihui Guo

**Affiliations:** State Key Laboratory of Crop Biology, College of Agronomy, Shandong Agricultural University, Tai’an 271018, China; diya_haixiaguo@163.com (H.G.); jinwangsdau@163.com (J.W.); xuehui@sdau.edu.cn (X.H.); ww_1999@126.com (X.C.); 15610418001@163.com (L.Z.); qixs3155@126.com (X.Q.); 18369503515@163.com (X.W.); gkoishi0514@gmail.com (J.L.); 13001755317@163.com (A.W.); ljl1005819@126.com (J.L.); cxy15621530169@126.com (X.C.); fczeng@sdau.edu.cn (F.Z.)

**Keywords:** proteomic and phosphoproteomic analyses, somatic embryogenesis, plant regeneration, cotton

## Abstract

Somatic embryogenesis (SE) is a biotechnological tool used to generate new individuals and is the preferred method for rapid plant regeneration. However, the molecular basis underlying somatic cell regeneration through SE is not yet fully understood, particularly regarding interactions between the proteome and post-translational modifications. Here, we performed association analysis of high-throughput proteomics and phosphoproteomics in three representative samples (non-embryogenic calli, NEC; primary embryogenic calli, PEC; globular embryos, GE) during the initiation of plant regeneration in cotton, a pioneer crop for genetic biotechnology applications. Our results showed that protein accumulation is positively regulated by phosphorylation during SE, as revealed by correlation analyses. Of the 1418 proteins that were differentially accumulated in the proteome and the 1106 phosphoproteins that were differentially regulated in the phosphoproteome, 115 proteins with 229 phosphorylation sites overlapped (co-differential). Furthermore, seven dynamic trajectory patterns of differentially accumulated proteins (DAPs) and the correlated differentially regulated phosphoproteins (DRPPs) pairs with enrichment features were observed. During the initiation of plant regeneration, functional enrichment analysis revealed that the overlapping proteins (DAPs-DRPPs) were considerably enriched in cellular nitrogen metabolism, spliceosome formation, and reproductive structure development. Moreover, 198 DRPPs (387 phosphorylation sites) were specifically regulated at the phosphorylation level and showed four patterns of stage-enriched phosphorylation susceptibility. Furthermore, enrichment annotation analysis revealed that these phosphoproteins were significantly enriched in endosomal transport and nucleus organization processes. During embryogenic differentiation, we identified five DAPs-DRPPs with significantly enriched characteristic patterns. These proteins may play essential roles in transcriptional regulation and signaling events that initiate plant regeneration through protein accumulation and/or phosphorylation modification. This study enriched the understanding of key proteins and their correlated phosphorylation patterns during plant regeneration, and also provided a reference for improving plant regeneration efficiency.

## 1. Introduction

Plants exhibit remarkable cellular totipotency [1,2,3], as demonstrated by somatic embryogenesis (SE) for plant regeneration. SE is a classic example of cellular totipotency and a promising biotechnological tool to generate new individuals. SE reduces the cost of plant propagation and provides a useful system for studying plant cell differentiation and development, totipotent expression, crop variety improvement, and mutant screening [4,5]. Plant regeneration is a process of totipotent expression in which somatic cells are reconstructed through dedifferentiation and redifferentiation, ultimately transforming into embryonic cells and resulting in the growth of a complete plant [4,6,7]. Although the regenerative ability of plants is widely used for the vegetative propagation of cultivated species, our current knowledge of its molecular background is limited [8].

Upland cotton (*Gossypium hirsutum* L.) is a pioneering crop for plant biotechnology in cell/genetic engineering and commonly achieves plant regeneration through the SE pathway. However, cotton is known for its weak ability to regenerate whole plants in culture, making it a notoriously recalcitrant species for plant regeneration [5,9,10]. Regarding tissue culture methods, cotton lags behind other major crops [11,12,13]. Cotton somatic embryos face several challenges, including genotype-dependent responses, high frequencies of abnormal embryo development, long culture cycles, low conversion rates into plants, lack of shoot elongation, and difficulties in rooting and browning [5,14]. These factors result in only a small percentage of somatic embryos maturing and regenerating into normal plants [15,16,17].

There is increasing evidence that various genes, proteins [18,19,20,21], endogenous phytohormones [22,23,24,25], and epigenetic factors [14,26,27,28] are involved in the process, including during the initiation of regeneration. However, data on the molecular basis of plant SE is still lacking. Furthermore, no comprehensive association analysis of proteins related to plant regeneration and their post-translational modifications has been reported.

A single proteome or phosphoproteome can usually only explain a single dimension of scientific problems in plant, but the association analysis of phosphorylated modification and protein expression can more accurately determine the dominant correlated regulatory mode. This combined analysis could reveal the differences/changes in protein expression and phosphorylated modification level and could jointly indicate the biological features in multiple dimensions. To explore the molecular basis underlying SE for plant regeneration at the proteomic and correlated post-translational modification levels, this study characterized an association of quantitative proteome and phosphoproteome databases. Our study focuses on two important stages of plant regeneration: callus embryogenic differentiation (PEC, primary embryogenic calli vs. NEC, non-embryogenic calli) and somatic embryo development (GE, globular embryos vs. PEC). We systematically analyzed the correlations between these phosphorylation events and changes in protein abundance using association analysis, and then identified differentially expressed key genes and pathways involved in plant regeneration processes. This information provides new insights into the impact of protein abundance and dynamic phosphorylation patterns on SE for plant regeneration.

## 2. Results

### 2.1. The Positive Correlation between Changes in Proteins and Their Phosphorylation

**Figure 1:**
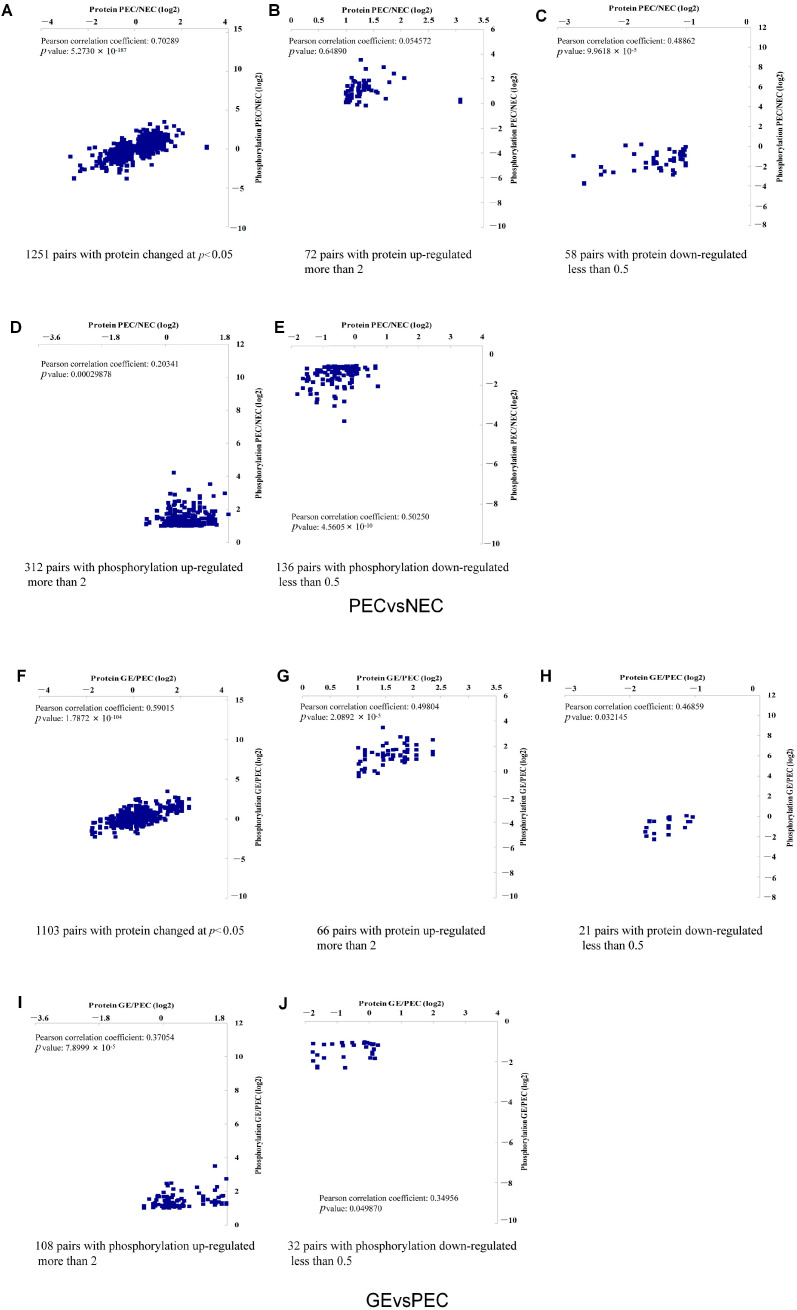
The positive correlation between changes in proteins and their phosphorylation. Correlation between protein and phosphorylated modification changes in PEC vs. NEC (**A**). Significantly upregulated proteins (**B**), significantly downregulated proteins (**C**), significantly upregulated phosphoproteins (**D**), and significantly downregulated phosphoproteins (**E**). Correlation between protein and phosphorylated modification changes in GE vs. PEC (**F**). Significantly upregulated proteins (**G**), significantly downregulated proteins (**H**), significantly upregulated phosphoproteins (**I**), and significantly downregulated phosphoproteins (**J**).

The molecular basis of somatic embryogenesis (SE) is still unclear, especially the callus embryogenic differentiation (PEC vs. NEC) and somatic embryo development (GE vs. PEC) processes. To determine the pattern characteristics of protein accumulation and phosphorylation regulation in the different periods during initiation of plant regeneration, an association analysis of the proteome and phosphoproteome was conducted during SE (Figure 1).

We analyzed the correlation between the quantitative proteome and phosphoproteome dataset obtained in our previous study. A total of 6730 proteins were identified in the proteome, whereas 2627 proteins (6301 sites) were identified in the phosphoproteome. Pearson’s correlation coefficients were calculated as 0.7, 0.59, and 0.67 in PEC vs. NEC (1251 pairs) (Figure 1A), GE vs. PEC (1103 pairs) (Figure 1F), and GE vs. NEC (1494 pairs) (Figure S1A), respectively. This was performed for all proteins significantly altered in terms of their phosphorylation, regardless of the direction of the change. Therefore, there is a positive correlation between the global proteome and phosphoproteome, indicating that the change pattern of the proteome is consistent with that of the phosphoproteome during SE. The analysis is restricted to the upregulated proteins and the downregulated proteins, as well as the upregulated and downregulated phosphorylated protein sites. These findings suggest that phosphorylation positively regulates protein accumulation during SE. The positive correlation observed between changes in protein levels and their phosphorylation status highlights the critical role of phosphorylation in regulating protein activity and stability.

### 2.2. Association Analysis of Differentially Accumulated Proteins (DAPs) and Correlated Differentially Regulated Phosphoproteins (DRPPs)

**Figure 2:**
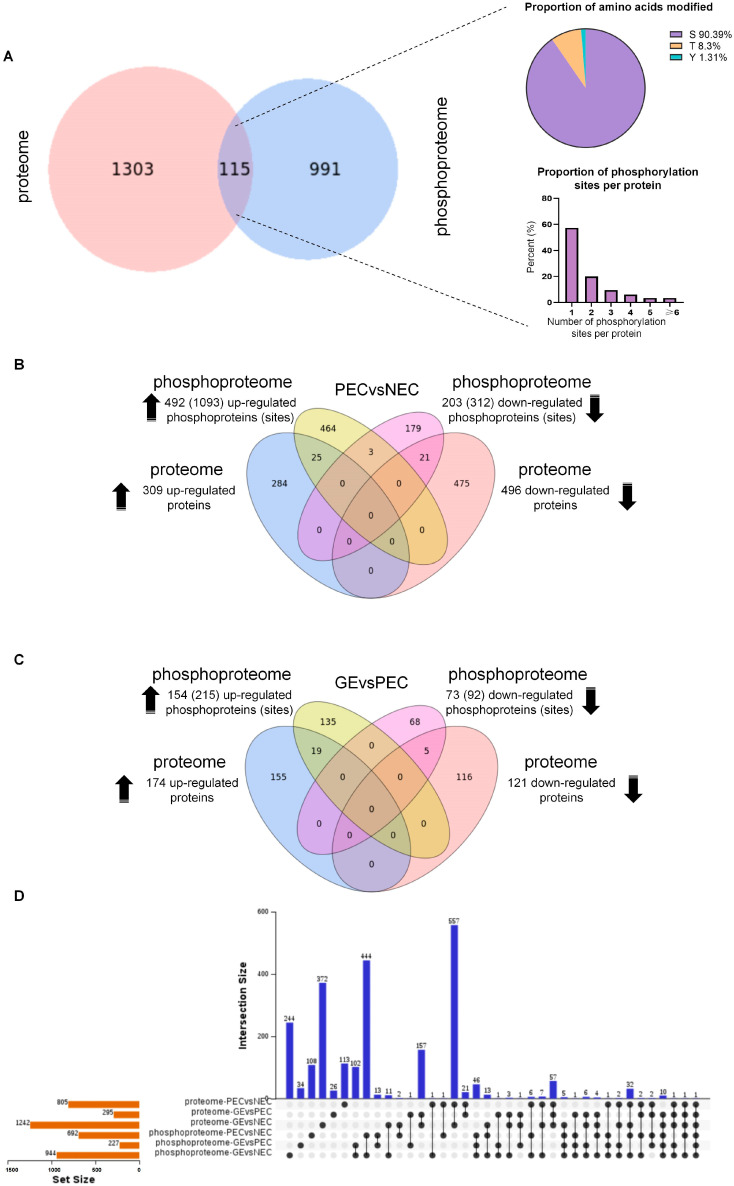
Association analysis of DAPs and correlated DRPPs during SE. (**A**) Venn diagram of DAPs and DRPPs in the proteome and phosphoproteome. The proportion of modified amino acids (upper right) and phosphorylation sites per protein (lower right) in overlapping proteins. S, serine; T, threonine; Y, tyrosine. (**B**) Venn diagram of up- and downregulated proteins/phosphoproteins in PEC vs. NEC. (**C**) Venn diagram of up- and downregulated proteins/phosphoproteins in GE vs. PEC. (**D**) Upset plots showing the intersection of DAPs and DRPPs of PEC vs. NEC, GE vs. PEC, and GE vs. NEC in the proteome and phosphoproteome. Blue bars: The horizontal axis represents comparison groups in different protein datasets (proteome or phosphoproteome), whereas the vertical axis represents the number of DAPs or DRPPs in each dataset. The red bars indicate the number of DAPs or DRPPs in different groups.

We identified 1418 DAPs and 1106 DRPPs (phosphorylation sites) in the 3 comparison groups (PEC vs. NEC, GE vs. PEC, and GE vs. NEC) with a fold change >2 in the proteome and phosphoproteome, respectively. Our analysis of DAPs and DRPPs from various datasets showed that 115 DRPPs (with 229 phosphorylation sites) overlapped with those in the proteome dataset. Proteins are mainly identified by serine modifications, with most proteins having only one phosphorylation site (Figure 2A). However, 3.48% of the phosphorylated proteins exhibited six or more modification sites, indicating more complex regulation during SE.

To characterize the overlapping proteins during callus embryogenic differentiation (PEC vs. NEC) and somatic embryo initiation (GE vs. PEC), Venn and Upset analyses were performed. A total of 492 (1093) phosphoproteins (sites) from the phosphoproteome overlapped with 309 proteins from the proteome by 25 (41) proteins (sites) (3.2%) among the upregulated proteins and phosphoproteins in PEC vs. NEC. The 203 (312) downregulated phosphoproteins (sites) overlapped with 496 downregulated proteins by 21 (33) proteins (sites) (3.1%) (Figure 2B). In GE vs. PEC, 19 (39) proteins (sites) (6.1%) that were upregulated overlapped with the upregulated phosphoproteins, and 5 (9) downregulated proteins (phosphorylation sites) (2.6%) overlapped (Figure 2C). Furthermore, we identified 68 (144) (5.6%) overlapping upregulated proteins and 27 (46) (3.0%) downregulated proteins, along with their corresponding phosphorylation sites, which overlap in GE vs. NEC (Figure S1F). This results indicate that during callus embryogenic differentiation and somatic embryo initiation, the upregulated proteins are more strongly responsive to phosphorylation regulation than the downregulated ones.

Our analysis combined DAPs and DRPPs from PEC vs. NEC, GE vs. PEC, and GE vs. NEC of the proteome and phosphoproteome, revealing a shared DAP/DRPP (A0A1U8IRL4: embryonic protein DC-8-like) during SE (Figure 2D). Meanwhile, the protein was upregulated in both proteomic and phosphoproteomic data of the SE development process, suggesting an important role for embryonic proteins during the initiation of plant regeneration. In addition, two DAPs-DRPPs, ECPP44-like phosphorylated proteins (A0A1U8JHR4 and A0A1U8MGI6) were identified, in which the proteome and phosphoproteome collectively exhibited PEC-specific patterns in PEC vs. NEC and GE vs. PEC.

### 2.3. Dynamic Trajectory Analysis of Overlapping Proteins (DAPs-DRPPs) during Plant Regeneration Initiation

**Figure 3:**
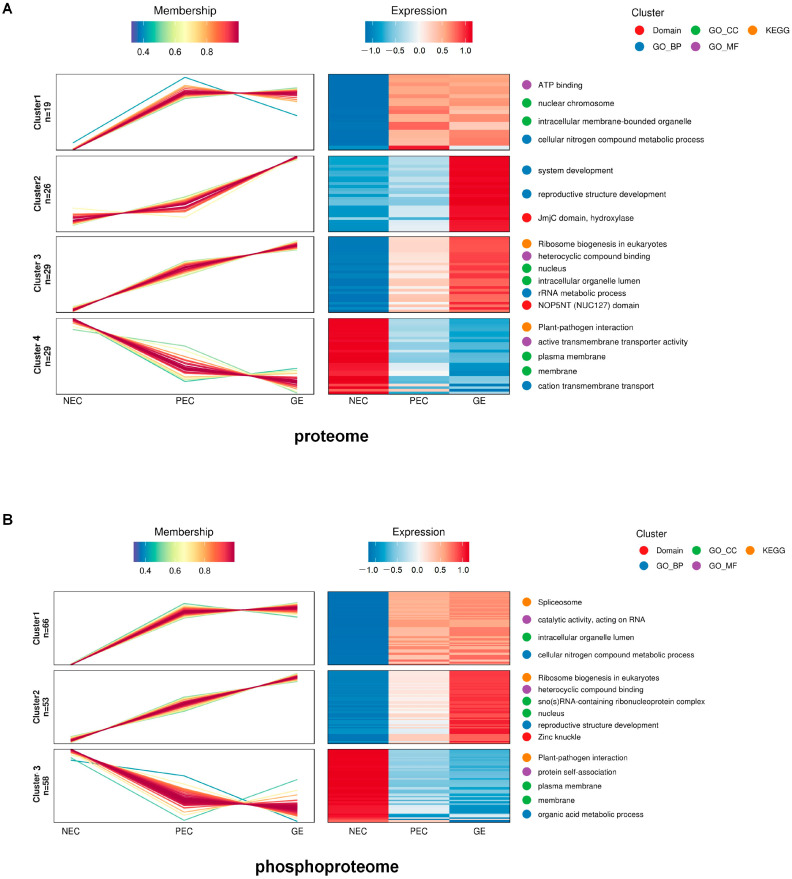
Hierarchical clustering analysis (HCA) of the overlapping proteins (DAPs-DRPPs) during plant regeneration initiation. (**A**) Hierarchical clustering analysis (HCA) of overlapping proteins in the quantitative proteome. Cluster identification and the number of profiles included in each cluster are indicated on the left. The *x*-axis represents the samples, and the *y*-axis represents the relative abundance of the proteins. The color of the line indicates the affiliation of the protein to the current class. Heat map: The *x*-axis represents the samples, the *y*-axis represents the different phosphoproteins, and the color of the heat map indicates the relative abundance of proteins in the sample. The two most significantly enriched items from the GO and Kyoto Encyclopedia of Genes and Genomes (KEGG) enrichment analyses are shown to the right of the corresponding cluster enrichment pattern clustering graphs. GO-CC: GO-cellular component. GO-BF, biological process. GO-MF: molecular function. (**B**) HCA of overlapping phosphoproteomic proteins. The *x*-axis represents the samples, and the *y*-axis represents the relative abundance of phosphoproteins.

To investigate the dynamic trajectory of overlapping proteins during SE, we performed hierarchical clustering analysis (HCA) on overlapping protein/phosphorylation sites in both the proteome and phosphoproteome. We identified seven major clusters significantly enriched in the proteome and phosphoproteome (Figure 3 and Table S1). Gene ontology (GO) term enrichment and Kyoto Encyclopedia of Genes and Genomes (KEGG) pathway enrichment analyses were conducted for each case (Figures S2 and S3).

The pattern of similarity between these clusters was considered to be a similar protein cluster pair. The cluster pair, proteome-cluster 1 (19 DAPs) and phosphoproteome-cluster 1 (29 DRPPs and 66 phosphorylation sites), shared 15 (39) proteins (phosphorylation sites) and were significantly enriched for GO biological processes (cellular nitrogen compound metabolic process). This indicates that protein synthesis and phosphorylation are crucial in regulating cellular nitrogen metabolism during regeneration initiation and embryo formation. The KEGG spliceosome pathway was significantly enriched in phosphoproteome-cluster 1 (Figure 3B and Figure S3D, and Table S1), indicating that phosphorylated proteins regulate the spliceosome pathway during SE, facilitating gene transcription.

Proteome-cluster 3 overlapped with 32 DRPPs (50 phosphorylation sites) from phosphoproteome-cluster 2. The pair of proteins showed considerable enrichment for GO biological processes, such as the development of reproductive structures, the development of the system, and postembryonic development, as well as the KEGG pathway for biogenesis in eukaryotes (Figure 3A,B, Figures S2C,D and S3C,D, and Table S1).

Proteome-cluster 4 (29 proteins) and phosphoproteome-cluster 3 (35 phosphoproteins and 58 phosphorylation sites) were the most similar protein cluster pair, with an overlap of 28 (49) proteins (phosphorylation sites). They were significantly enriched for GO biological processes, specifically cation transmembrane transport (Figure 3A,B, Figure S2C and S3C, and Table S1).

Therefore, we hypothesized that the overlapping proteins in the three pairs of HCA clusters represented three gene classes involved in SE regulation with different patterns. This result also confirms the positive correlation between protein accumulation and phosphorylation.

### 2.4. Enrichment Analysis of Proteins Susceptible to Regulation by Phosphorylation

**Figure 4:**
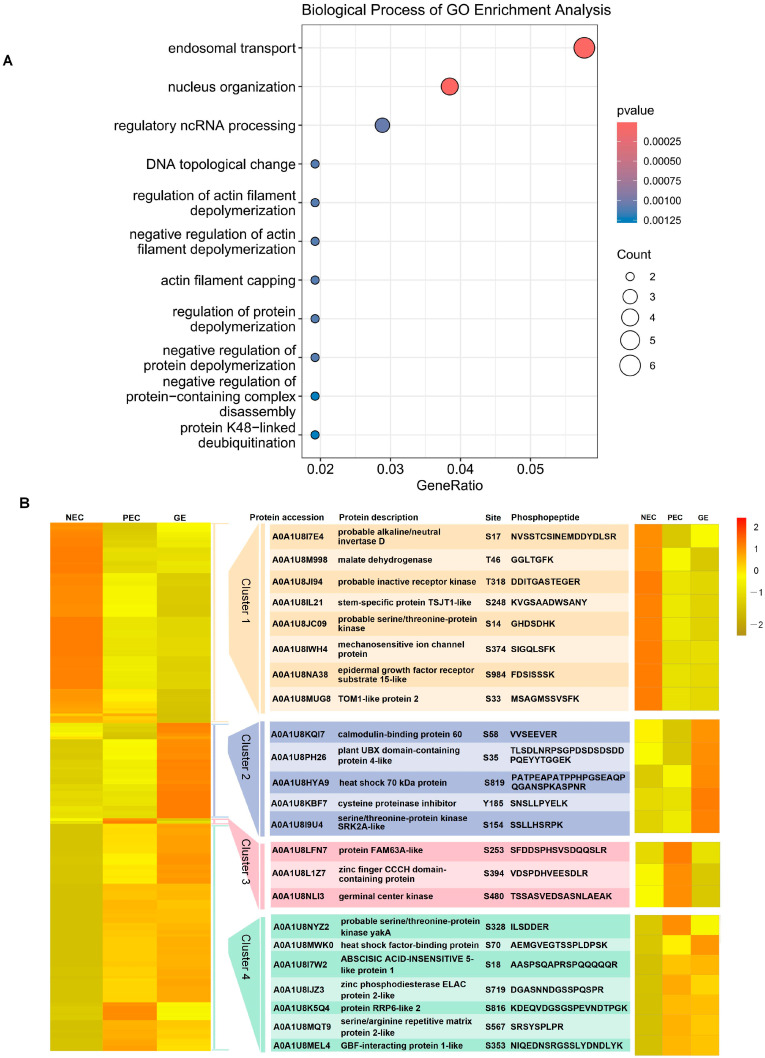
Proteins susceptible to regulation by phosphorylation during SE. (**A**) GO enrichment analysis of the proteins susceptible to regulation by phosphorylation. (**B**) Heat map of the 198 phosphoproteins (387 sites). A global color gradient ranging between green for proteins with fold change ≤ 0.5, yellow for values between 0.6 and 2, and red for values ≥ 2.0 was applied to the clustered data set.

To investigate the molecular basis of phosphorylation regulation, we performed an enrichment analysis of proteins that did not accumulate significantly in the proteome but had significant changes (fold change > 2) in the phosphoproteome. A total of 198 phosphoproteins (387 sites) (Table S3) were differentially regulated only at the phosphorylation level. GO enrichment analysis revealed that these phosphoproteins were significantly enriched in endosomal transport and nucleus organization processes (Figure 4A).

In addition, we analyzed four major phosphorylated protein clusters based on their protein site enrichment characteristics. Trends in the phosphorylation patterns are shown in Figure 4B as a heatmap. The figure shows 23 representative phosphorylated proteins from 4 phosphorylation clusters. Upregulated phosphoproteins in clusters 1, 2, and 3 were significantly enriched during the NEC, GE, and PEC periods, respectively. Phosphoproteins in phosphorylation cluster 4 were upregulated to varying degrees during both PEC and GE compared to NEC (Figure 4B). Proteins showed stage-enriched susceptibility to phosphorylation regulation during SE, as indicated by the results. The phosphorylation pattern was significantly altered. Furthermore, three proteins showed significant differential regulation based solely on their phosphorylation levels: FAM63A-like (A0A1U8LFN7), zinc-finger CCCH domain-containing protein (A0A1U8L1Z7), and germinal center kinase (A0A1U8NLI3). These proteins exhibited similar expression patterns during the critical period of regeneration initiation (Figure 4B).

## 3. Conclusions

Based on the above results, this study reveals association pattern of proteome and phosphoproteome during plant regeneration initiation. The strong correlation between changes in protein expression level and phosphorylation status underscores the importance of post-translational modifications. The analysis of overlapping proteins and their dynamic trajectories reveals a coordinated molecular response critical for plant regeneration. The key signaling pathways discovered in this study are the core of phosphorylation mediated regulation. These insights not only deepen our understanding of plant regeneration but also open avenues for improving regenerative processes.

## 4. Discussion

Vogel [1], Xu et al. [18], and Yu et al. [29] have all contributed to the compelling scientific puzzle of somatic cell totipotency in plant regeneration and its challenging problem in biology. How plants initiate the somatic-to-embryonic transition remains unclear. The molecular basis for the initiation of regeneration is also unclear, particularly in systematic studies of protein accumulation and modification. This study systematically characterized the quantitative proteome and phosphoproteome during important representative periods in cotton, a pioneer crop for genetic biotechnology applications. The aim was to obtain a better understanding of the molecular basis underlying SE regulation for plant regeneration. Such studies are of fundamental and practical importance for plant cell engineering and biotechnological breeding.

### 4.1. DAPs-DRPPs with Different Trajectory Patterns during Plant Regeneration Initiation

This study identified seven major clusters in the proteome and phosphoproteome with significant enrichment features (Figure 3). Three similar pairs of protein enrichment trajectories and the corresponding three classes of DAP-DRPPs with different trajectory patterns from the seven protein clusters in the proteome and phosphoproteome were summarized (Table S2). As SE progressed, class 1 proteins exhibited a pattern of abrupt surges, followed by a flat or slight slope, from the NEC to the PEC to the GE. This protein cluster was significantly and simultaneously enriched in the term ‘cellular nitrogen compound metabolic process’, and nitrogen metabolism-related proteins play a crucial role in protein synthesis and amino acid supply. They are involved in the processes of synthesis, catabolism, and translocation of amino acids to meet the nitrogen source requirements for plant growth and development [30,31]. Therefore, our study suggests that the up-slope enrichment pattern of these proteins may be a significant characteristic of plant regeneration initiation. Furthermore, in class 2, DAPs-DRPPs from proteome clusters 2 and 3, as well as phosphoproteome-cluster 2, were upregulated linearly. These clusters were primarily enriched in GO biological processes, such as reproductive structure development, system development, and postembryonic development, as well as KEGG pathways, such as ribosome biogenesis in eukaryotes. These findings suggest a potential positive regulation of embryogenic differentiation, particularly in the formation of somatic embryos. Proteome-cluster 4 and phosphoproteome-cluster 3 exhibited an opposite pattern to classes 1 and 2. Changes in phosphorylated protein levels can disrupt the balance of hormones involved in SE. Therefore, the downregulation of phosphorylated proteins may indicate a shift in cellular resources and signaling priorities toward defense responses.

The combined analysis of proteomic and phosphoproteomic data has unveiled new insights into the temporal regulation of protein during regeneration. For example, specific proteins have been observed to undergo changes in expression and phosphorylation states at different stages of regeneration. These changes ensure the dynamic regulation of proteins required for tissue differentiation during regeneration.

### 4.2. Proteins Susceptible to Regulation by Phosphorylation

The process of protein phosphorylation regulates cell proliferation, development, differentiation, signal transduction, etc. Plant SE is a complex process that involves various regulatory mechanisms, including protein phosphorylation. Therefore, it suggested that phosphorylation-mediated signaling could be considered as an important aspect in plant regeneration.

Our study focused on 198 DRPPs susceptible to phosphorylation regulation and exhibited variable phosphorylation patterns. We conducted a comparative analysis of representative developmental periods (NEC, PEC, and GE) of SE (Figure 4B; Table S3). Cluster 1 proteins were downregulated and enriched during the PEC and GE periods, whereas cluster 4 exhibited a contrasting phosphorylation pattern to cluster 1. This suggests that both types of proteins may have distinct regulatory roles in the acquisition and maintenance of embryonic competence. Several DRPPs, such as probable alkaline/neutral invertase D, probable serine-/threonine-protein kinase, TOM1-like protein 2, ABSCISIC ACID-INSENSITIVE 5-like protein 1, and protein RRP6-like 2, are involved in sucrose catabolism, signal transduction, protein transportation, phytohormone signaling, and epigenetic regulation, respectively [32,33,34,35,36]. The serine/arginine repetitive matrix protein 2-like (A0A1U8MQT9) showed 26 phosphorylation sites in PEC and GE, with the highest number of phosphorylation sites among these 198 DRPPs. This protein belongs to the RNA recognition motif (RRM) superfamily, which regulates post-transcriptional gene expression [37], but has not been described in detail in plants. In addition, studies have shown that ABA biosynthesis is an important step in establishing auxin-induced cell totipotency [38,39]. In cluster 2, we observed proteins susceptible to phosphorylation, including calmodulin-binding protein 60, heat shock 70 kDa protein, cysteine proteinase inhibitor, and serine/threonine-protein kinase SRK2A. Previous study has found that calcium signal is an important factor regulating auxin-induced callus and revealed a molecular pathway through which calcium signal and auxin signal interact to regulate plant regeneration and development [40]. Their phosphorylation sites were significantly enriched during the GE period. These proteins may play important roles in the regulation of various biological processes through phosphorylation.

In addition, the previous results identified three proteins that exhibited significant differential regulation based solely on their phosphorylation levels. The protein FAM63A-like is enriched during the initiation of embryogenic differentiation and is annotated as a member of the evolutionarily conserved and structurally unique MINDY-1 deubiquitinase family, as well as a K48-linked deubiquitination enzyme [41]. Therefore, FAM63A-like likely plays a crucial role in protein phosphorylation by removing ubiquitin modifications from proteins tagged with K48-linked chains [42]. Furthermore, proteins containing the zinc-finger CCCH domain play a crucial role in plant development and stress response [43,44] and function in metal ion and mRNA binding [41]. The zinc-finger CCCH domain-containing protein 17-like is enriched during the initial stages of embryogenic differentiation, suggesting it may be involved in this critical developmental process. It is likely to regulate gene expression patterns, mRNA processing, and other molecular events necessary for the establishment and progression of SE. The isoform X2 of germinal center kinase 1-like is also enriched during the embryogenic differentiation stage. This protein contains a protein kinase domain that regulates signaling pathways during plant development through protein phosphorylation [45]. As a protein kinase, it phosphorylates target proteins, thus regulating their activity, stability, subcellular localization, and interactions with other molecules. Germinal center kinase 1-like isoform X2 may contribute to the coordination of cellular processes and gene expression programs required for successful embryogenic development by modulating signaling pathways. More research is necessary to identify their specific substrates, interaction partners, and underlying mechanisms to understand their functional implications in plant SE and their potential applications in crop improvement and stress responses.

### 4.3. Potential Critical DAPs-DRPPs in the Initiation of Plant Regeneration

More and more studies have found that many important life activities are not only related to the abundance of proteins, but more importantly are regulated by various protein post-translational modifications. Protein phosphorylation is one of the most common and important covalent modifications in vivo. This study also explores the role of protein phosphorylation in plant regeneration, focusing on the key signaling pathways and molecular players involved.

The association analysis between DAPs and DRPPs reveals a significant overlap, indicating that many proteins undergoing changes in abundance are also subject to differential phosphorylation. This dual regulation suggests a coordinated mechanism where changes in protein levels are fine-tuned by phosphorylation to achieve precise control over cellular functions. It is worth noting that previous studies preliminarily identified the phosphorylated protein ECPP44 and proposed its potential role in embryogenic capacity [46,47]. Stress processing experiments ultimately showed that it was phosphorylated during the transition to embryogenesis. More research is required to understand the specific functions of ECPP44 during SE. This could involve studying its interactions with other proteins, examining its role in stress responses and signaling pathways, and determining its contribution to embryogenic competence.

Protein phosphorylation plays a crucial role in plant regeneration by regulating key signaling pathways essential for tissue reprogramming. Advances in association analysis of proteomics and phosphoproteomics have significantly enhanced our understanding of the dynamic changes in protein expression and phosphorylation during regeneration. These insights into the signaling pathways of phosphorylation-dependent regulation provide a valuable knowledge base for improving regeneration ability through targeted biotechnological approaches.

## 5. Materials and Method

### 5.1. Association Analysis of the Proteome and Phosphoproteome

Based on the quantitative results of the proteome obtained in previous study [48] and the phosphoproteome data that will be made public, we calculate the Pearson correlation between the two in R (version 4.3.2) [49]. Pearson’s correlation coefficient (r), a statistical measure of the strength of a linear relationship between paired data, was used for this analysis. Furthermore, r is constrained between +1 and −1. A positive coefficient indicates a positive correlation, a negative coefficient indicates a negative correlation, and a coefficient of 0 indicates no correlation. The correlation between variables is indicated by the sign of the correlation coefficient. The closer the coefficient is to +1 or −1, the stronger the correlation [50].

### 5.2. Analysis of DAPs and Correlated DRPPs (Differential Protein Statistics)

To identify the overlapping and specific proteins in PEC vs. NEC, GE vs. PEC, and GE vs. NEC, we performed Venn diagrams and Upset plot in both the proteome and phosphoproteome using ggplot2 (version 3.4.3).

### 5.3. Hierarchical Clustering Analysis

We performed hierarchical clustering analysis (HCA) on differentially phosphorylated sites based on their relative phosphorylation intensity. This analysis, using the Mfuzz R package (version 2.44) [51], employed a cluster number (k) of 6 and a fuzzification parameter (m) of 2.

### 5.4. Functional Enrichment Analysis

Gene annotation of the proteome was derived from the UniProt database (www.uniprot.org). Proteins were categorized based on their GO annotations into three groups: biological processes, cellular compartments, and molecular functions. The enrichment of differentially modified proteins within each category was evaluated using a two-tailed Fisher exact test against all identified proteins, with a significance level of *p* < 0.05.

To identify enriched pathways, the KEGG database was utilized. A two-tailed Fisher’s exact test was conducted to assess the enrichment of differentially modified proteins compared to all identified proteins. Pathways with a corrected *p*-value < 0.05 were considered statistically significant. These pathways were then categorized into hierarchical groups according to the KEGG website.

The results and figures for GO enrichment and KEGG pathway analysis were generated using the clusterProfiler package (version 4.10.1) [52].

### 5.5. Enrichment-Based Clustering

For further hierarchical clustering based on differentially modified protein functional classification (such as GO, domain, pathway, and complex), we first collated all the categories obtained after enrichment along with their *p* values, and then filtered for those categories which were at least enriched in one of the clusters with *p* value < 0.05. This filtered *p* value matrix was transformed by the function x = −log10 (*p* value). Finally, these x values were z-transformed for each functional category. These z scores were then clustered by one-way hierarchical clustering (Euclidean distance, average linkage clustering) in Genesis. Cluster membership was visualized using the “heatmap.2” function from the “gplots” package (version 3.1.3.1).

## Data Availability

The original contributions presented in the study are included in the article/Supplementary Material, further inquiries can be directed to the corresponding author.

## Supplementary Materials

The following supporting information can be downloaded at: https://www.mdpi.com/article/10.3390/genes15081079/s1, Figure S1: Comparative integrated analysis of DAPs and DRPPs in GE vs. NEC. Correlation between protein and phosphorylated modification changes in GE vs. NEC (A). Significantly upregulated proteins (B), significantly downregulated proteins (C), significantly upregulated phosphoproteins (D), and significantly downregulated phosphoproteins (E). (F) Venn diagram of up- and downregulated proteins/phosphoproteins in GE vs. NEC; Figure S2: GO and KEGG enrichment analysis of DAPs in HCA clusters. (A–C) Gene ontology (GO) enrichment analysis of individual clusters in Figure 3A. GO-CC, GO-cellular component. GO-MF, molecular function. GO-BF, biological process. (D) Kyoto Encyclopedia of Genes and Genomes (KEGG) enrichment plots of each cluster in Figure 3A; Figure S3: GO and KEGG enrichment analysis of DRPPs in HCA clusters. (A–C) GO enrichment analysis of the individual cluster in Figure 3B. GO-CC, GO-cellular component. GO-MF, molecular function. GO-BF, biological process. (D) KEGG enrichment plots of individual clusters in Figure 3B; Table S1: Annotation of DAP and DRPP clusters for HCA analysis; Table S2: Three pairs of DAPs-DRPPs clusters were involved in SE; Table S3: Proteins susceptible to regulation by phosphorylation.
