# Supplementary material for: Proteomic and Phosphoproteomic Analyses during Plant Regeneration Initiation in Cotton (Gossypium hirsutum L.)
Source: Genes (Basel). 2024 Aug 15;15(8):1079. doi: 10.3390/genes15081079 (PMC11353933; doi:10.3390/genes15081079)
Supplement: Supplementary file 1 [file genes-15-01079-s001.zip › Supplementary Figures_Jul 25, 2024.zip!/Supplementary Figures_Jul 25, 2024.pdf]

Figure S1

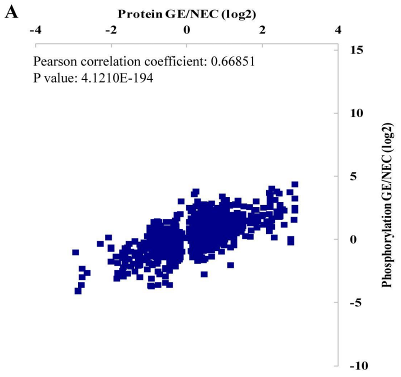

1494 pairs with protein changed at p<0.05

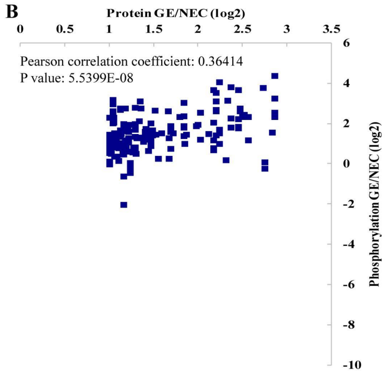

210 pairs with protein up-regulated more than 2

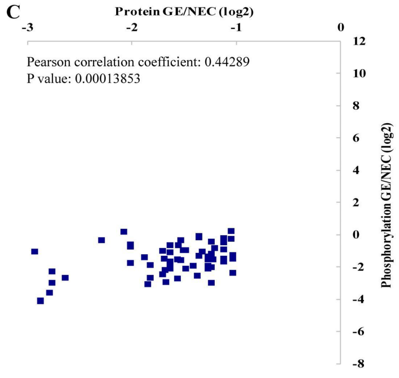

69 pairs with protein down-regulated less than 0.5

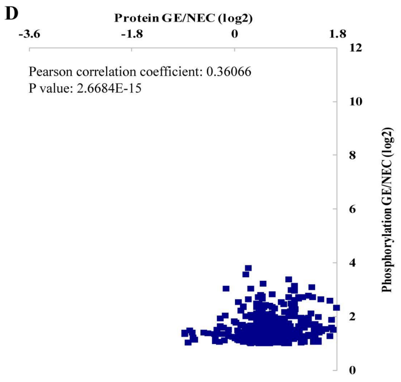

451 pairs with phosphorylation up-regulated more than 2

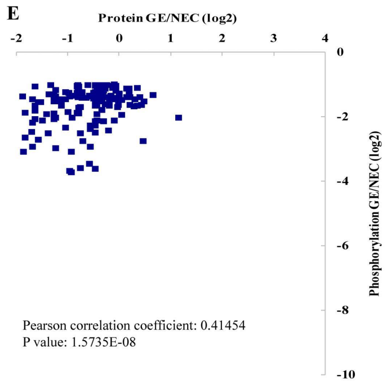

172 pairs with phosphorylation down-regulated less than 0.5

GEvsNEC

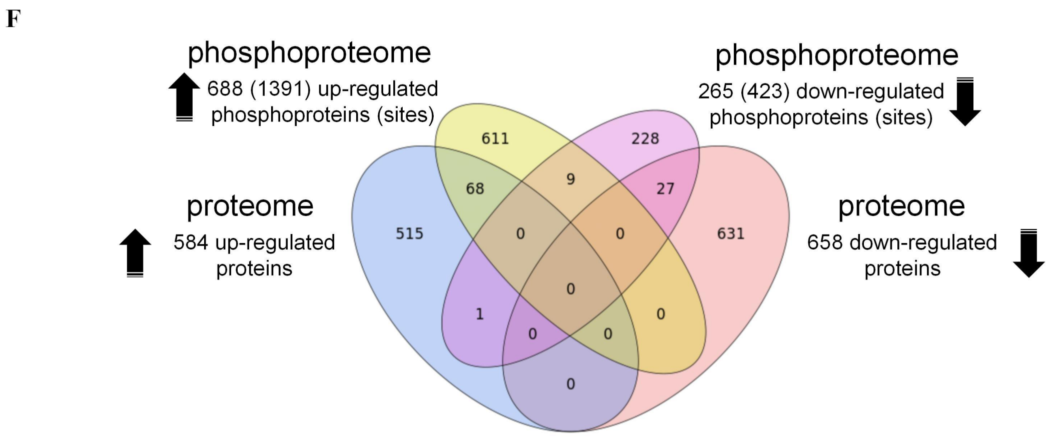

GEvsNEC

Figure S2

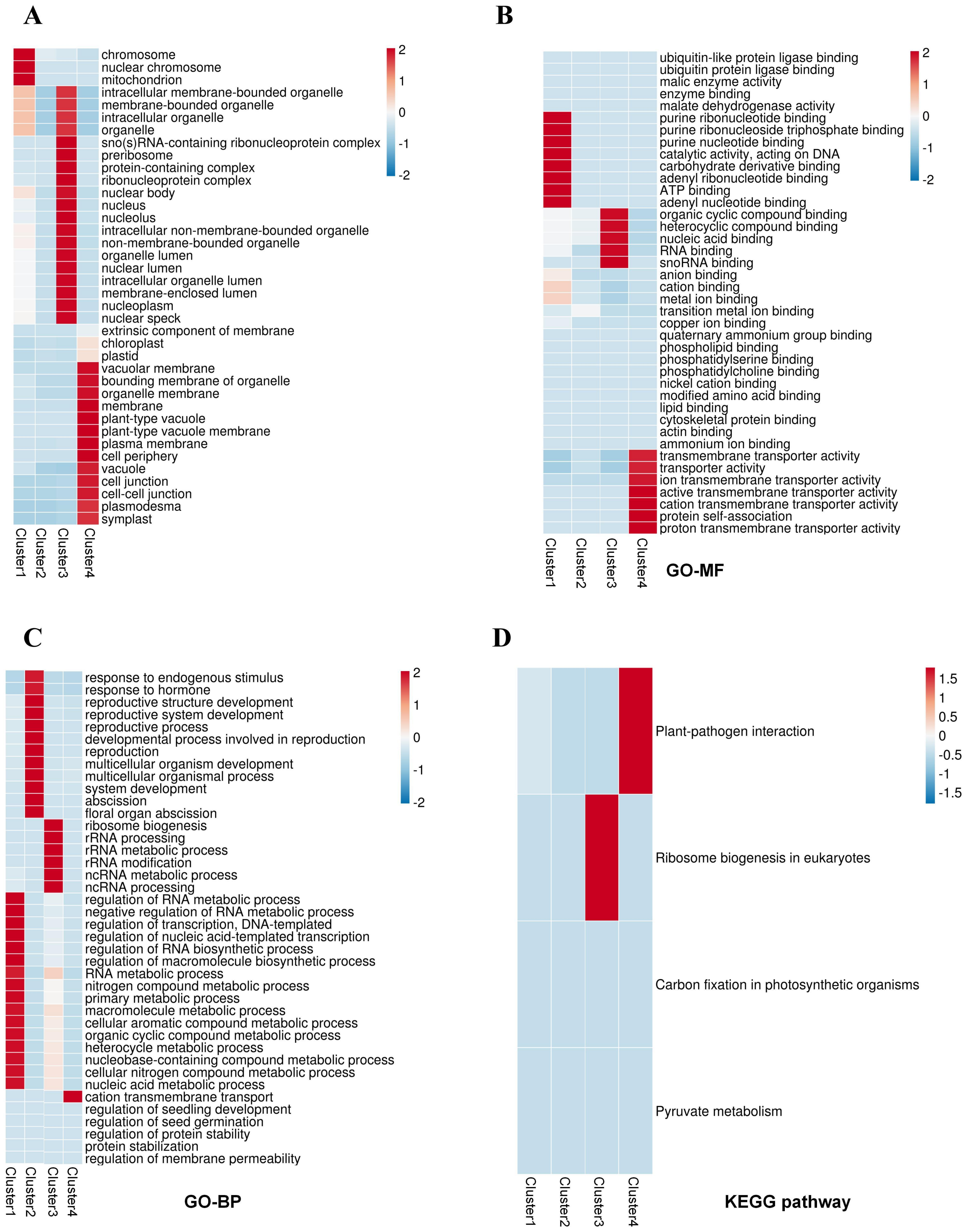
A
chromosome
nuclear chromosome
mitochondrion
intracellular membrane-bounded organelle
membrane-bounded organelle
intracellular organelle
organelle
sno(s)RNA-containing ribonucleoprotein complex
preribosome
protein-containing complex
ribonucleoprotein complex
nuclear body
nucleus
nucleolus
intracellular non-membrane-bounded organelle
non-membrane-bounded organelle
organelle lumen
nuclear lumen
intracellular organelle lumen
membrane-enclosed lumen
nucleoplasm
nuclear speck
extrinsic component of membrane
chloroplast
plastid
vacuolar membrane
bounding membrane of organelle
organelle membrane
membrane
plant-type vacuole
plant-type vacuole membrane
plasma membrane
cell periphery
vacuole
cell junction
cell-cell junction
plasmodesma
symplast
Cluster1
Cluster2
Cluster3
Cluster4
2
1
0
-1
-2
B
ubiquitin-like protein ligase binding
ubiquitin protein ligase binding
malic enzyme activity
enzyme binding
malate dehydrogenase activity
purine ribonucleotide binding
purine ribonucleoside triphosphate binding
purine nucleotide binding
catalytic activity, acting on DNA
carbohydrate derivative binding
adenyl ribonucleotide binding
ATP binding
adenyl nucleotide binding
organic cyclic compound binding
heterocyclic compound binding
nucleic acid binding
RNA binding
snoRNA binding
anion binding
cation binding
metal ion binding
transition metal ion binding
copper ion binding
quaternary ammonium group binding
phospholipid binding
phosphatidylserine binding
phosphatidylcholine binding
nickel cation binding
modified amino acid binding
lipid binding
cytoskeletal protein binding
actin binding
ammonium ion binding
transmembrane transporter activity
transporter activity
ion transmembrane transporter activity
active transmembrane transporter activity
cation transmembrane transporter activity
protein self-association
proton transmembrane transporter activity
Cluster1
Cluster2
Cluster3
Cluster4
GO-MF
2
1
0
-1
-2
C
response to endogenous stimulus
response to hormone
reproductive structure development
reproductive system development
reproductive process
developmental process involved in reproduction
reproduction
multicellular organism development
multicellular organismal process
system development
abscission
floral organ abscission
ribosome biogenesis
rRNA processing
rRNA metabolic process
rRNA modification
ncRNA metabolic process
ncRNA processing
regulation of RNA metabolic process
negative regulation of RNA metabolic process
regulation of transcription, DNA-templated
regulation of nucleic acid-templated transcription
regulation of RNA biosynthetic process
regulation of macromolecule biosynthetic process
RNA metabolic process
nitrogen compound metabolic process
primary metabolic process
macromolecule metabolic process
cellular aromatic compound metabolic process
organic cyclic compound metabolic process
heterocycle metabolic process
nucleobase-containing compound metabolic process
cellular nitrogen compound metabolic process
nucleic acid metabolic process
cation transmembrane transport
regulation of seedling development
regulation of seed germination
regulation of protein stability
protein stabilization
regulation of membrane permeability
Cluster1
Cluster2
Cluster3
Cluster4
GO-BP
2
1
0
-1
-2
D
Plant-pathogen interaction
Ribosome biogenesis in eukaryotes
Carbon fixation in photosynthetic organisms
Pyruvate metabolism
Cluster1
Cluster2
Cluster3
Cluster4
KEGG pathway
1.5
1
0.5
0
-0.5
-1
-1.5

Figure S3

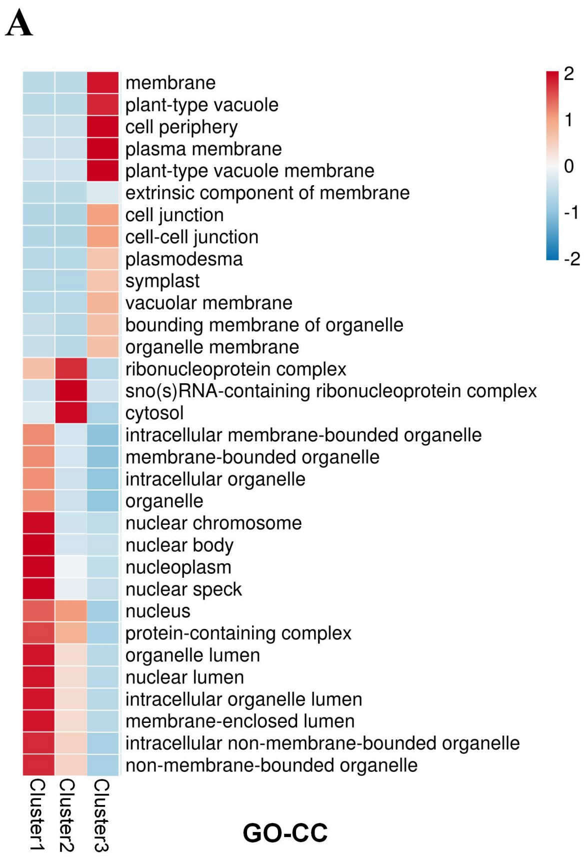
A
membrane
plant-type vacuole
cell periphery
plasma membrane
plant-type vacuole membrane
extrinsic component of membrane
cell junction
cell-cell junction
plasmodesma
symplast
vacuolar membrane
bounding membrane of organelle
organelle membrane
ribonucleoprotein complex
sno(s)RNA-containing ribonucleoprotein complex
cytosol
intracellular membrane-bounded organelle
membrane-bounded organelle
intracellular organelle
organelle
nuclear chromosome
nuclear body
nucleoplasm
nuclear speck
nucleus
protein-containing complex
organelle lumen
nuclear lumen
intracellular organelle lumen
membrane-enclosed lumen
intracellular non-membrane-bounded organelle
non-membrane-bounded organelle
Cluster1
Cluster2
Cluster3
2
1
0
-1
-2
GO-CC

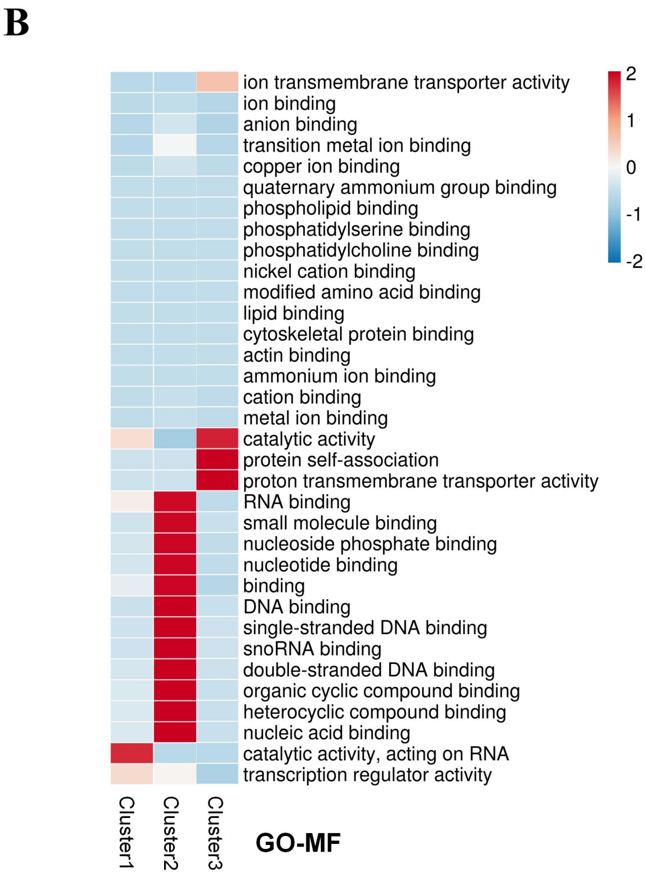
B
ion transmembrane transporter activity
ion binding
anion binding
transition metal ion binding
copper ion binding
quaternary ammonium group binding
phospholipid binding
phosphatidylserine binding
phosphatidylcholine binding
nickel cation binding
modified amino acid binding
lipid binding
cytoskeletal protein binding
actin binding
ammonium ion binding
cation binding
metal ion binding
catalytic activity
protein self-association
proton transmembrane transporter activity
RNA binding
small molecule binding
nucleoside phosphate binding
nucleotide binding
binding
DNA binding
single-stranded DNA binding
snoRNA binding
double-stranded DNA binding
organic cyclic compound binding
heterocyclic compound binding
nucleic acid binding
catalytic activity, acting on RNA
transcription regulator activity
Cluster1
Cluster2
Cluster3
2
1
0
-1
-2
GO-MF

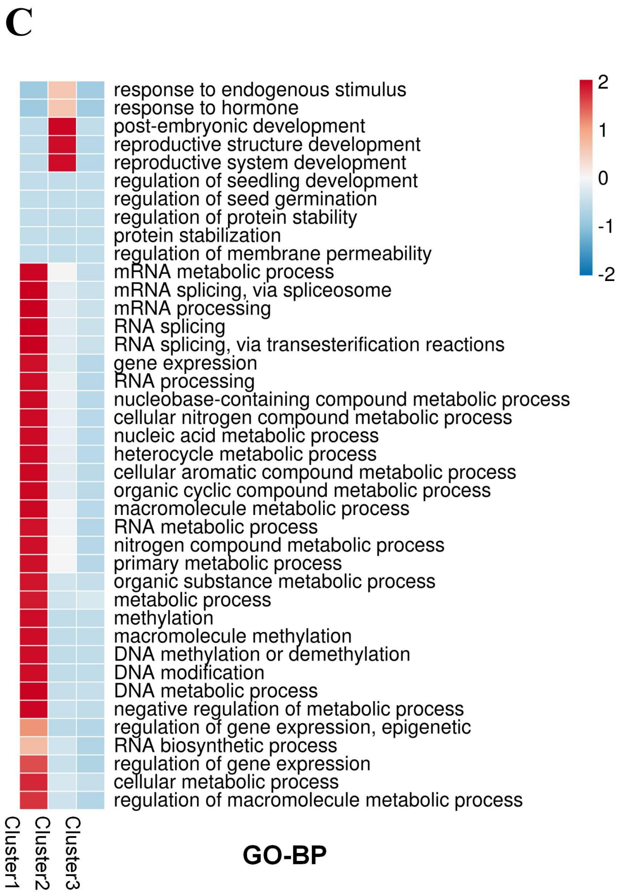
C
response to endogenous stimulus
response to hormone
post-embryonic development
reproductive structure development
reproductive system development
regulation of seedling development
regulation of seed germination
regulation of protein stability
protein stabilization
regulation of membrane permeability
mRNA metabolic process
mRNA splicing, via spliceosome
mRNA processing
RNA splicing
RNA splicing, via transesterification reactions
gene expression
RNA processing
nucleobase-containing compound metabolic process
cellular nitrogen compound metabolic process
nucleic acid metabolic process
heterocycle metabolic process
cellular aromatic compound metabolic process
organic cyclic compound metabolic process
macromolecule metabolic process
RNA metabolic process
nitrogen compound metabolic process
primary metabolic process
organic substance metabolic process
metabolic process
methylation
macromolecule methylation
DNA methylation or demethylation
DNA modification
DNA metabolic process
negative regulation of metabolic process
regulation of gene expression, epigenetic
RNA biosynthetic process
regulation of gene expression
cellular metabolic process
regulation of macromolecule metabolic process
Cluster1
Cluster2
Cluster3
2
1
0
-1
-2
GO-BP

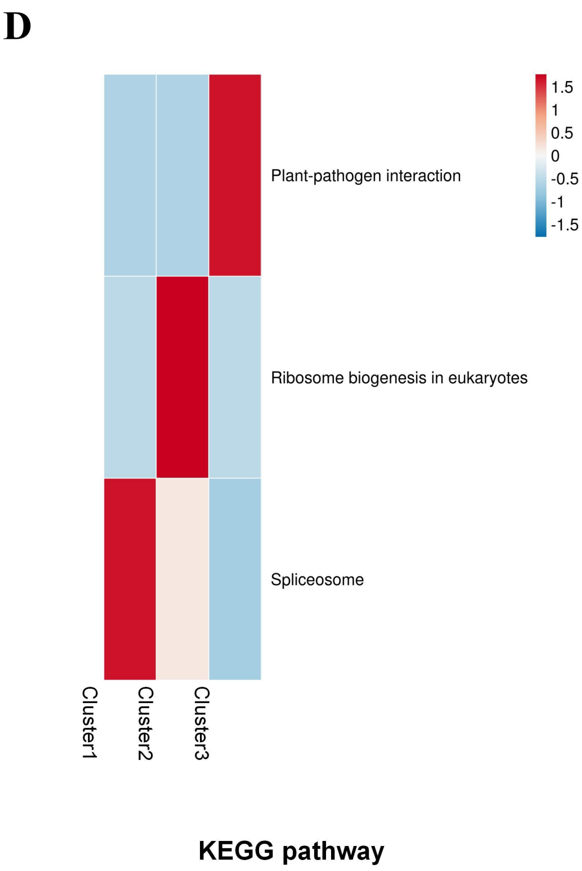
D
Plant-pathogen interaction
Ribosome biogenesis in eukaryotes
Spliceosome
Cluster1
Cluster2
Cluster3
1.5
1
0.5
0
-0.5
-1
-1.5
KEGG pathway
